# Climate Change as a Social Determinant of Health: An Interactive Case-Based Learning Activity

**DOI:** 10.15766/mep_2374-8265.11332

**Published:** 2023-08-02

**Authors:** Arindam Sarkar, Nital P. Appelbaum, Rathi Asaithambi, Connie Tran, Doris Lin, Anjali Aggarwal, Stephanie Nguyen

**Affiliations:** 1 Assistant Professor, Department of Family and Community Medicine, Baylor College of Medicine; 2 Assistant Professor, Department of Education, Innovation and Technology, and Assistant Dean, Medical Education Research and Scholarship, Baylor College of Medicine; 3 Assistant Professor, Department of Pediatrics, Baylor College of Medicine; 4 Associate Professor, Department of Anesthesiology, Baylor College of Medicine; 5 Associate Professor, Department of Medicine, Baylor College of Medicine; 6 Associate Professor, Department of Family and Community Medicine, Baylor College of Medicine

**Keywords:** Environmental Determinants of Health, Case-Based Learning, Community-Based Medicine, Population Health, Social Determinants of Health, Climate Change, Diversity, Equity, Inclusion

## Abstract

**Introduction:**

Despite consensus on climate change's impact on humans, medical schools have not widely adopted inclusion of environmental topics into their mandatory curriculum. This educational activity explicitly addresses climate change as one of the environmental determinants of health (EDH).

**Methods:**

We developed a required, 1-hour module for all first-year medical students. This interactive, case-based, small-group activity was incorporated into a curriculum within an advising program but could be run independently. Before and after the session, participants completed evaluations assessing knowledge gains and attitude shifts.

**Results:**

Of 183 first-year students, 155 completed both pre- and postmodule surveys. Participants' rating increased on the postmodule survey item “priority should be given to the discussion of EDH in medical education.” The Wilcoxon signed rank test determined this difference in priority was statistically significant (*p* < .001). Reported strengths of this activity included the cases, informative content on EDH, the video, the discussion, and highlighted EDH resources. Suggested areas for improvement included more information on how to apply concepts to clinical contexts, guidance on how to engage in EDH concepts, and more discussion time. As a result of the module, students planned to engage in recycling, reduced consumption, advocacy, and changes to mode of transportation.

**Discussion:**

Climate change remains the greatest global threat to human health, and future physicians must be equipped to educate patients and policymakers on the harms of environmental hazards. This brief yet effective module offers one approach to incorporating this topic into medical school curricula.

## Educational Objectives

By the end of this activity, learners will be able to:

1. Identify several direct and indirect effects of climate change on human health.
2. Recognize the unequal burden of environmental hazards on vulnerable and marginalized communities (with lower socioeconomic status, ends of the age spectrum, or communities of color).
3. Summarize the actions students can take to mitigate detrimental environmental effects on patients.

## Introduction

Although climate change has been consistently identified as one of the greatest threats to humans, medical school curricula have very little coverage of its health consequences.^1^ In a 2020 survey of medical schools across 118 countries, only 16% included climate change in their curricula.^2^ In another study of a dozen U.S. medical schools, although 84% of students believed climate change should be included in the core curriculum, only 13% of students felt their school provided adequate education.^3^ While at least 20 U.S. medical schools have affirmed a commitment to educating learners on climate topics by joining the Global Consortium on Climate and Health Education, most schools offer only optional electives.^4^ After a review of *MedEdPORTAL,* we found one resource addressing climate change utilizing a standardized patient to educate students on wildfires causing asthma exacerbations.^5^ At the time of writing, however, no published studies have demonstrated effective class-wide integration and incorporation of climate topics into the required curriculum for all students.

One school found that a 6-week environmental health module combining didactic and experiential elements significantly increased medical students' self-reported sense of preparedness to discuss environmental health issues with their patients.^6^ A growing number of leaders in medical education agree that climate topics are essential to prepare future physicians to address issues such as health inequities, sustainability, and environmental justice.^7,8^ The following module on environmental determinants of health (EDH) aimed to address gaps in students' knowledge and attitudes regarding climate change and human health. EDH collectively refer to any physical, chemical, or biological factors external to a person that influence human health.^9^ Global, national, and local environmental behaviors represent one aspect of EDH. EDH may overlap with social determinants of health under the greater common theme of nonmedical drivers of health.

## Methods

All Baylor College of Medicine (BCM) medical students were assigned an advisor within the Learning Community (LC) program. In addition to general professional advising, the LC program required all first-year students to participate in recurring small-group sessions (eight students) facilitated by their LC advisor. We incorporated this EDH module as one of the sessions and leveraged the 24 existing advisors to serve as small-group faculty facilitators. The time commitment required by the advisor-facilitators was minimal and included a 30-minute review led by the module leaders prior to the 1-hour small-group session.

Although we utilized our LC advisors to serve as facilitators, an LC program was not necessary as the module could be easily incorporated into any part of a medical school curriculum with minimal and even self-directed training of facilitators. Moreover, small groups were not required as the module could also be taught in a large-group setting. The module could be run independently, with the cases minorly modified to fit any student level of clinical exposure. Our participants were second-semester, preclinical students with a basic understanding of history taking and simple patient counseling.

The module consisted of a video introduction (Appendix A)^10^ and a facilitated small-group discussion. A single-occurrence, 1-hour session was felt to be an appropriate setting for this pilot program aimed at exploring the effects of climate change and its disproportionate impact on vulnerable populations.

### EDH Module Development

On the 2020–2021 annual evaluation of the LC program, many students reported an interest in climate change and environmental topics. Those comments provided the initial impetus for this EDH module. Four LC faculty with expertise in medical education and preventative medicine developed this 1-hour activity. A group of second-, third-, and fourth-year students offered feedback on the case vignettes to improve their relevance and engagement for the target audience.

A facilitator guide (Appendix B) was distributed to all 24 LC faculty to introduce the topic of environmental health. This guide provided a link to a short video from the Centers for Disease Control and Prevention (CDC), module objectives, recommended time cues, and cases with discussion questions. The facilitator guide and a walk-through of the EDH module were presented during a regularly scheduled monthly LC advisor meeting. This hour-long orientation session led by the module developers allowed other facilitators to familiarize themselves with the material and brainstorm personalized ways to promote discussions within their regularly assigned student cohorts.

### Module Implementation

After reminding students to complete the preactivity survey (Appendix C), facilitators began the EDH module by reviewing the session's objectives and role within the larger curriculum. The module continued with a statement of ground rules reminding students to speak freely but respectfully, keep opinions confidential, and listen nonjudgmentally to their peers. Next, the video from the CDC (Appendix A) was viewed to introduce the health-related consequences of climate change.^10^ Students were subsequently introduced to three case presentations highlighting specific environmental hazards such as asthma triggers, heat stress, and vector-borne diseases. Students were encouraged to read the cases aloud, with the facilitator posing the discussion questions. Each group was given freedom to customize the discussion and dialogue based on individual group dynamics. Time stamps throughout the guide prompted facilitators on when to advance to the next section of the module. The discussion concluded by reviewing take-home messages and allowing students time to complete the postmodule survey (Appendix D).

Two supplemental references were included at the end of the facilitator guide (Appendix B) if students or faculty were interested in optional further reading. The first item was a summary of the physical evidence of climate change by the Intergovernmental Panel on Climate Change.^11^ The second was an overview by the CDC of region-specific direct and indirect effects of climate change.^12^ Analogous reports for any U.S. region were available on the same website.

An important objective of this module was to emphasize to students that the impacts of climate change were not equally distributed across the U.S. population. The discussion questions of case 2 addressed this theme. The provided clinical example of heat stress affecting a manual laborer prompted the facilitator to ask learners to describe other examples of environmental hazards to vulnerable individuals.

Beyond acquisition of knowledge on how environmental factors might impact future patients' health, the module also encouraged students to become aware of relevant local resources and engage in advocacy efforts. In case 1, facilitators queried students about familiarity with the air quality index on smartphones to counsel patients with sensitive respiratory illnesses. In case 3, students were prompted to explore avenues to personally reduce carbon emissions and practice resource conscientiousness.

### Pre- and Postmodule Evaluation Surveys

To evaluate whether the EDH module achieved its objectives, students received a premodule survey (Appendix C) and a postmodule survey (Appendix D) via SurveyMonkey. BCM's Institutional Review Board approved these two questionnaires and the overall educational activity. The preactivity survey contained three items: (a) a question on the perceived level of priority that should be given to environmental health topics in medical education, (b) a knowledge assessment question on the effects of climate change, and (c) a question to gauge current engagement with environmental conservation, climate change advocacy, and/or medical resource conscientiousness.

The postmodule survey contained the same knowledge assessment and prioritization questions. Its third item queried intended engagement in future environmental conservation, climate change advocacy, and/or medical resource conscientiousness. Considering the ordinal nature of our priority Likert item and its non-normal distribution, the Wilcoxon signed rank test was used to test for paired differences.^13^ Two additional free-text items offered students the opportunity to provide feedback on the strengths and areas for improvement of the overall activity.

The surveys assessed lower levels of Kirkpatrick's pyramid (reaction: text comment on module strengths and areas for improvement; learning: pre/post change in prioritization, pre/post knowledge item for Educational Objective 1, intention to engage in EDH).^14^ Not all objectives were directly assessed by the survey instrument due to concerns about survey length and fatigue at our school. Educational Objectives 2 and 3 were formatively assessed by facilitators during the session through targeted case-based discussion prompts. To optimize survey participation, small-group facilitators were instructed to reserve 5 minutes prior to the start of the module and 5 minutes at the end of the module for students to complete the surveys.

Premodule and postmodule surveys were connected for within-person analyses on assessment items, along with overall counts for text responses. We used content analysis to identify patterns for the text comments. One faculty member proposed emerging patterns, and another cross-checked findings to arrive at a consensus. No additional steps were required to complete the content analysis as consensus was reached.

## Results

Of 183 total first-year students, 155 (85% paired response rate) completed both pre- and postmodule evaluation surveys. In addition, 14 students completed only the premodule survey, and six students completed only the postmodule survey.

### Reaction and Learning

The Wilcoxon signed rank test indicated that the median postmodule survey ranks for priority given to EDH in medical education (*Mdn* = 4.0) were statistically significantly higher than the median premodule survey ranks (*Mdn* = 3.0, *Z* = −4.44, *p* < .001; Table 1). While there was slight improvement in postmodule survey knowledge scores, the vast majority of respondents answered correctly on both the pre- and postmodule surveys (Table 2).

**Table 1.**
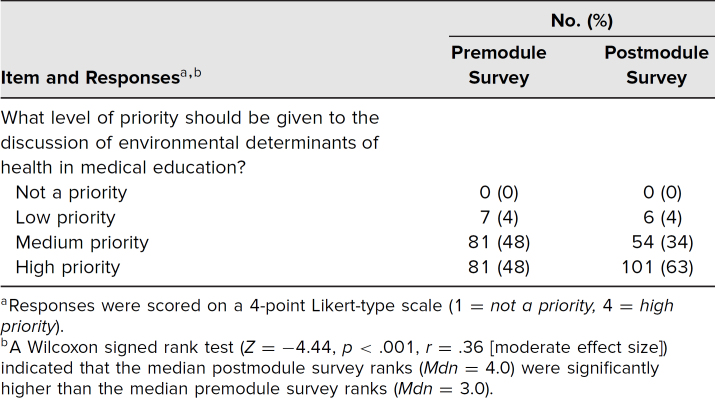
Perceived Priority of Environmental Determinants of Health

**Table 2.**
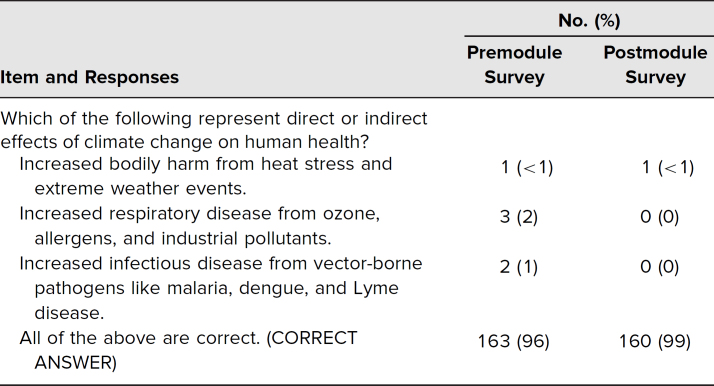
Knowledge Assessment Item

Of 122 text comments, the top emerging patterns on strengths of the module were the cases/examples (34 comments), the video (23 comments), informative content (22 comments), relevant content (20 comments), and the discussion component (17 comments; Table 3).

**Table 3.**
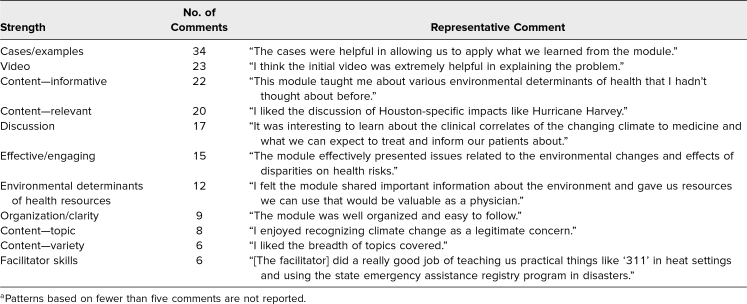
Text Comment Patterns on Module Strengths (*N* = 122 Comments)^a^

Of 70 comments on areas for improvement, top suggestions were more information on how to apply concepts to patient/health care context (12 comments), guidance on how to engage in EDH concepts in their current roles (12 comments), additional cases/examples (seven comments), and more discussion time (seven comments; Table 4).

**Table 4.**
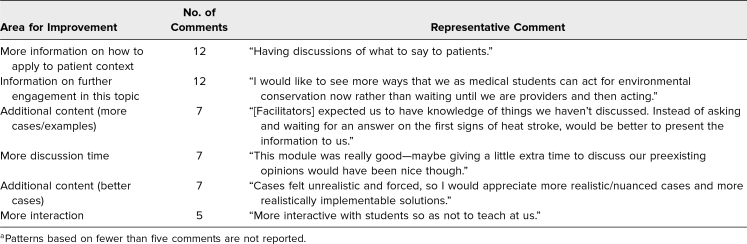
Text Comment Patterns on Module Areas for Improvement (*N* = 70 Comments)^a^

### Engagement

On the premodule survey, the majority of students (*n* = 110, 65%) were not engaged in environmental conservation, climate change advocacy, and/or medical resource conscientiousness but broadly planned to do so in the future. Of the 14% of respondents (*n* = 24) who were engaged, main activities included recycling (eight comments), lessened personal impact on environment (seven comments), and dietary changes (four comments).

On the postmodule survey, 31 students (19%) indicated that, as a result of the module, they would engage in a form of environmental conservation, climate change advocacy, and/or medical resource conscientiousness. Examples of behaviors provided via text comment included recycling (eight comments), reduced consumption (eight comments), advocacy (five comments), and changes to mode of transportation (five comments). The majority of respondents did not plan to engage immediately following the module but planned to engage in such activities in the future (*n* = 102, 63%). Representative comments from this group included “Try to educate patients and the community, get involved with advocacy and politics,” “Use my platform as a physician in the future to advocate for environmental sustainability,” and “Stay more informed on negative environmental health effects specific to the city I am practicing in.”

## Discussion

This module was conceived, developed, and evaluated with student input at every stage. Through the existing framework of LC small groups, 24 clinical faculty delivered a novel, interactive, case-based module to teach first-year medical students the impacts of climate change on human health. We feel this activity bolstered student awareness of climate change's relevance to medicine. It also reinforced an interconnectedness that all health care professionals can share through resource conscientiousness and sustainability efforts. Based on student feedback, the module appears to have successfully introduced our early clinical learners to EDH. Although we implemented the EDH module within our LC program, medical schools can adapt it into any part of their curriculum or deliver the content as a stand-alone module.

The survey data suggest that this EDH module positively influenced students' rating of the curricular priority for EDH in medical education. Several participants commented that they specifically enjoyed how the module identified methods they could immediately employ to mitigate environmental hazards. These tools included using their smartphone weather app to educate patients about air quality indexes and becoming familiar with their local county's heat plan and resources. Several students also reported interest in learning more about EDH via an upcoming elective course.

This educational exercise constitutes a brief, unique, and customizable curriculum to address a gap in undergraduate medical education. The learning objectives are specific and align with the cases and discussion questions. The module development process and faculty training are feasible and reproducible. The module covers EDH with varied examples of climate change within a 60-minute session. The facilitator guide provides an intuitive overview of EDH in a relevant and digestible format. The video introduction and cases are succinct and practical. Furthermore, the case discussion questions embrace social constructivism and encourage participants to engage with the educational objectives by collaboratively learning from one another.

Although this resource provides a framework for addressing climate change and EDH, some elements have limited generalizability. For example, one of the cases uses data and resources that are region specific. Additionally, the activity was piloted at one institution and with one class of medical students. Modifying the module based on student input from multiple institutions and classes would strengthen future iterations.

Selecting a knowledge assessment question of appropriate difficulty was another challenge. With a very high correct-response rate on the premodule survey, there was little room to demonstrate improvement and knowledge gain. While respondents may not have necessarily recognized the implication of each answer choice, the availability of “all of the above” likely provided an easy default option that required students to recognize only two correct options. A pilot screening of the survey questions before implementing the case-based learning activity was not performed. Nonetheless, we feel the use of this item helped to reinforce for participants the primary objective of identifying several examples of pervasive and well-evidenced effects of climate change. For future iterations, we have increased the difficulty level for the knowledge assessment question.

Furthermore, although the case discussion prompts addressed Educational Objectives 2 and 3, the pre/post surveys did not directly assess these items. Future offerings of the postactivity survey will directly assess Educational Objective 2 by asking students to identify an example of how EDH disproportionally impact financially disadvantaged communities and Educational Objective 3 by asking students to identify any planned behavior changes to incorporate resource conscientiousness.

Students identified a desire for concrete guidance on how they could engage in EDH concepts now, as well as how to integrate such concepts into their future patient care. Future iterations of this curriculum could be implemented across the educational continuum and provide more guidance on steps to increase EDH engagement. For example, instead of providing students with resources, cases could encourage students to discover tools such as the air quality index themselves and practice utilizing resources with simulated patients.

Teaching about the impacts of climate change poses unique challenges to medical educators. Unlike an individual organ system or disease process, environmental medicine topics can range from heat-related illness and vector-borne diseases to natural disasters and air pollution. Beyond the historical politicization of renewable energy and conservation, diverse individual climate topics are not easily incorporated into a traditional course on the molecular origins of disease and organ function. Although this publication reflects one iteration of an educational activity, we feel it represents an essential step in a paradigm shift for our curriculum. We recognize that more is needed to gain competence on climate change's role in medicine. As integrated members and leaders of their greater communities, our graduates developed an awareness and understanding of the necessity of taking action.

The current pandemic notwithstanding, climate change remains the greatest threat to global health in the 21st century.^15^ This novel initiative to incorporate EDH into the mandatory curriculum for all students will better equip them to practice in an ever-changing health care landscape.

## Appendices


How Climate Affects Community Health.mp4Facilitator Guide.docxPremodule Survey.docxPostmodule Survey.docx
*All appendices are peer reviewed as integral parts of the Original Publication.*
