# Supplementary material for: Climate Change as a Social Determinant of Health: An Interactive Case-Based Learning Activity
Source: MedEdPORTAL. 2023 Aug 2;19:11332. doi: 10.15766/mep_2374-8265.11332 (PMC10394120; doi:10.15766/mep_2374-8265.11332)
Supplement: Supplementary file 1 — How Climate Affects Community Health.mp4Facilitator Guide.docxPremodule Survey.docxPostmodule Survey.docx [file mep_2374-8265.11332-s001.zip › C. Premodule Survey.docx]

Environmental Determinants of Health (EDH) Module

*Pre-Module Survey*

This survey contains items that are designed to help us evaluate the quality of the inaugural Environmental Medicine module. You will receive pre- and post-module survey requests. All responses are confidential and will be aggregated to create summary reports. Data collected from this survey will be used to improve future years’ module and curriculum around Environmental Medicine. Your participation is appreciated.

1. What level of priority should be given to the discussion of environmental determinants of health in medical education?

- 1. High priority
  2. Medium priority
  3. Low priority
  4. Not a priority

2. Which of the following represent direct or indirect effects of climate change on human health?

- 1. Increased bodily harm from heat stress and extreme weather events
  2. Increased respiratory disease from ozone, allergens and industrial pollutants
  3. Increased infectious disease from vector-borne pathogens like malaria, dengue and Lyme disease
  4. All of the above are correct
  5. None of the above are correct

3. I currently engage in a form of environmental conservation, climate change advocacy and/or medical resource conscientiousness.

- 1. Strongly Disagree
  2. Disagree
  3. Neither Agree or Disagree
  4. Agree
  5. Strongly Agree
