# Supplementary material for: Climate Change as a Social Determinant of Health: An Interactive Case-Based Learning Activity
Source: MedEdPORTAL. 2023 Aug 2;19:11332. doi: 10.15766/mep_2374-8265.11332 (PMC10394120; doi:10.15766/mep_2374-8265.11332)
Supplement: Supplementary file 1 — How Climate Affects Community Health.mp4Facilitator Guide.docxPremodule Survey.docxPostmodule Survey.docx [file mep_2374-8265.11332-s001.zip › D. Postmodule Survey.docx]

Environmental Determinants of Health (EDH) Module

*Post-Module Survey*

1. What level of priority should be given to the discussion of environmental determinants of health in medical education?

- 1. High priority
  2. Medium priority
  3. Low priority
  4. Not a priority

2. Which of the following represent direct or indirect effects of climate change on human health?

- 1. Increased bodily harm from heat stress and extreme weather events
  2. Increased respiratory disease from ozone, allergens and industrial pollutants
  3. Increased infectious disease from vector-borne pathogens like malaria, dengue and Lyme disease
  4. All of the above are correct
  5. None of the above are correct

3. As a result of the Environmental Medicine module, I intend to engage in a form of environmental conservation, climate change advocacy and/or medical resource conscientiousness.

- 1. Strongly Disagree
  2. Disagree
  3. Neither Agree or Disagree
  4. Agree
  5. Strongly Agree

4. Please comment on any STRENGTHS of the module. [Text box]

5. Please comment on any AREAS FOR IMPROVEMENT for the module. [Text box]
