# Supplementary material for: Climate Change as a Social Determinant of Health: An Interactive Case-Based Learning Activity
Source: MedEdPORTAL. 2023 Aug 2;19:11332. doi: 10.15766/mep_2374-8265.11332 (PMC10394120; doi:10.15766/mep_2374-8265.11332)
Supplement: Supplementary file 1 — How Climate Affects Community Health.mp4Facilitator Guide.docxPremodule Survey.docxPostmodule Survey.docx [file mep_2374-8265.11332-s001.zip › B. Facilitator Guide.docx]

Environmental Determinants of Health (EDH) Module

*Facilitator Guide*

1. **Module Objectives**
   1. Identify several direct and indirect effects of climate change on human health
   2. Recognize the unequal burden of environmental hazards on vulnerable and marginalized communities (with lower socioeconomic status, ends of the age spectrum, or communities of color)
   3. Summarize the actions students can take to mitigate detrimental environmental effects on patients
2. **Competency Goals**
   1. Medical Knowledge: Utilize the principles of public health, epidemiology, and biostatistics of climate change in identifying and reducing the incidence, prevalence, and severity of disease to improve health.
   2. Patient Care: Utilize critical thinking and assess health risks of environmental factors to develop a prioritized problem list and differential diagnosis and to incorporate environmental health counseling in patient care.
   3. Practice-based learning and improvement: Applying principles and practices of evidence-based medicine of climate change in making decisions about prevention, diagnosis and treatment of disease.
   4. Leadership: Demonstrate the ability to work effectively as a member of an interprofessional health care team to advocate for recognition of the effects of climate change on health and investments in health care infrastructure in preparation for the health effects of climate change.

*[15 minutes]* Provide 5 minutes for students to complete the Pre-Module survey. Then review Ground Rules and watch Video.

1. **Ground Rules**
   1. Environmental medicine is a diverse, complex, and potentially divisive topic. Today, we’ll focus on what content you’ll need as a physician to manage and prevent the impact of climate change on your patients. We’ll also discuss what you as a medical student can start doing now.
   2. You are encouraged to share but not required. As always, we remind ourselves about the privacy and confidentiality of small group discussions.
   3. We’ll reserve 5 minutes at the end of this module to complete a post-module survey. This is the first year of this activity and your input will help improve the discussion for future students.
2. **Video**

a. [CDC - How Climate Affects Community Health](https://www.youtube.com/watch?v=JywsWktvODc)^[[1]](#endnote-1)^ [4 minutes, 37 seconds]

*[35 minutes]* The following three cases contain broader discussion topics independent of the vignette. Feel free to draw from your own experiences of medical waste, legislative advocacy, or ecological conservation.

1. **Cases**
   1. Case 1: Alba Uterol is an 8-year-old girl who presents to your outpatient clinic for a well-child check. Last year, she was wheezing twice a week but now it’s every day. She’s not exercising more than before, but she is generally more sniffly and sneezy. Alba’s family recently moved to an industrial part of town, where her father works at a nearby chemical plant.
      1. What are some factors that could be affecting Alba’s breathing?
         1. Alba’s worsening breathing could be asthma disease progression or a result of increased airborne pathogens and pollutants.
         2. Urban air pollution is primarily due to airborne particulate matter, nitrogen dioxide, volatile organic compounds, and ozone.^[[2]](#endnote-2)^ Most of these pollutants are in highest concentration where petrochemical refineries and manufacturing plants exist.
         3. The Asthma and Allergy Foundation of America reports that since 2010, respiratory allergens like hay weed, pollen and poison ivy are experiencing longer active seasons (due to higher surface temperatures) and wider geographic influence (due to drought and changing transcontinental wind patterns).^[[3]](#endnote-3)^
         4. Black, Hispanic, and Indigenous communities in the U.S. experience more asthma-related hospitalizations (due to physical environments, tobacco use, access to healthcare, and other social determinants of health).^[[4]](#endnote-4)^
      2. What is ozone (O_3_) in relation to air pollution?
         1. “Good” ozone occurs naturally in the upper atmosphere where it blocks harmful UV radiation. “Bad” ozone forms at ground level when industrial pollutants react to form photochemical smog.
         2. In areas with high levels of “bad” ozone, communities experience more asthma attacks, pneumonias, cardiovascular damage, increased susceptibility to infections and decreased lung function.
         3. Hospital admissions, ER visits and asthma exacerbations occur more commonly on “Ozone Days” with elevated ground ozone levels. Harris County and many others report when they are experiencing “Ozone Days”.^[[5]](#endnote-5)^
         4. Even a few unhealthy ozone days a year cause measurable harm. Houston has dozens of annual Ozone Days.^[[6]](#endnote-6)^
         5. Your smartphone’s Weather app has a daily Air Quality Index (AQI) you can educate patients on. On days with unhealthy AQI (greater than 100 on a scale of 0-500), susceptible patients can be recommended to stay indoors or avoid strenuous activity.
   2. Case 2: Rob DiMayo is a 35-year-old man who presents to the emergency room with 4 hours of malaise, headache, and dark urine. He started feeling unwell while at work outdoors doing roofing. In the hospital, he was found to have a creatine kinase >1500 and a creatinine >1.5.
      1. What are some direct and indirect consequences of rising surface temperatures?
         1. Directly, rising surface temperatures are associated with increased heat stress, soil degradation, desertification, loss of biodiversity, degradation of freshwater ecosystems, and depletion of stratospheric ozone
         2. Indirectly, there’s increased physical injuries during natural disasters [e.g., floods, hurricanes, wildfires] and malnutrition during famines. Interestingly, a mental health link has been established between warmer summers and increased violence^[[7]](#endnote-7)^
      2. Has anyone heard of the Heat Stress continuum?
         1. Heat Rash/Prickly Heat
            1. Symptoms: Cluster of red pimples or blisters on neck, chest or groin
            2. Management: Keep the rash dry with powder and don’t use ointments that impair cooling
         2. Heat Cramps
            1. Symptoms: Spasms or pain in the abdomen, arms or legs
            2. Management: Drink more fluids to avoid electrolyte derangements (K, Mg, Ca) which can cause cardiac problems
         3. Heat Syncope
            1. Symptoms: Fainting, dizziness
            2. Management: Drink more fluids as with cramps
         4. Heat Exhaustion
            1. Symptoms: Headache, nausea, decreased urine output
            2. Management: Evaluate in ER, cold compresses, ice bath
         5. Heat Stroke
            1. Symptoms: Confusion, seizures, slurred speech, profuse sweating
            2. Management: Call 911, cold packs to head, neck, groin
      3. What populations are most impacted by increases in heat stress? What can we do?
         1. Individuals with outdoor occupations may be at greater risk of negative health consequences of extreme heat. Persons affected by food insecurity, lack of transportation or reliable housing are also prone to environmental hazards like heat stress. These factors are known as social vulnerability – which overlap with more commonly known social determinants of health.
         2. Globally, communities with lower incomes experience the brunt of adverse health outcomes. The WHO estimates between 2030 and 2050, climate change will cause 250,000 additional deaths per year not only from heat stress but also from malnutrition, malaria, diarrhea, and dengue.^[[8]](#endnote-8)^
         3. From 2003-2008, there were 263 deaths reported in Texas from exposure to excessive natural heat.^[[9]](#endnote-9)^ The average annual Texas surface temperature in 2036 is expected to be 3.0°F warmer than the 1950-1999 average and 1.8°F warmer than the 1991-2020 average. The number of 100-degree days is expected to double by 2036 compared to 2001-2020.^3^
         4. The City of Houston has Cooling Centers, usually housed at libraries, recreation centers, multi-service centers and other facilities that are open to the public. When the City’s Heat Plan is in effect (posted on houstonemergency.org) METRO provides free transportation to designated cooling centers. Residents who need transportation can call 311 to request service.
      4. Are there other examples of certain populations being disproportionate are impacted by environmental hazards?
         1. Children born prematurely or with underdeveloped immune systems may have increased sensitivity to airborne allergens. Persons with asthma or chronic obstructive pulmonary disease may be more sensitive to air pollution. Older adults with limited mobility and persons with chronic medical conditions are more vulnerable to health stressors, such as extreme heat, floods, poor air quality, and other climate-related events.
   3. Case 3: Mary Alarya is a 41-year-old woman admitted to the hospital for fever to 104, headache, myalgias and jaundice. You order a blood smear and microscopy which reports visualization of Giemsa-stained parasites.
      1. Why are cases of mosquito-borne illnesses like malaria, dengue and Zika dramatically increasing in nonendemic places like the U.S.?
         1. Warmer surface temperatures present more favorable conditions for the survival, reproduction and completion of the life cycle of vectors like Aedes mosquitos and Ixodes ticks.^1^
         2. Worsening flooding creates stagnant mosquito breeding waters and changes in riverbed ecologies of tsetse flies.^1^
         3. Cases of other infectious diseases are also rising. Desertification and drought cause affected populations to consume poorer quality drinking water (e.g., increased cases of leptospirosis, campylobacter infections and cryptosporidiosis)^1^
      2. What actions are governments and institutions taking to lessen carbon emissions?
         1. Texas is the national leader in wind energy production. 1/5 of our state’s energy comes from renewable sources like wind and solar^3^
         2. Compared to data from 2007, the U.S. produces 43 times as much solar energy now and the average American uses 10% less energy now.^[[10]](#endnote-10)^
         3. The majority of carbon emissions stem from commercial energy needs, transportation, industry, and agriculture. China generates around 30% of all global emissions and the U.S. generates 14%.^[[11]](#endnote-11)^
         4. Some students may feel personal changes are useless and stricter regulations for corporations at the national and international level are paramount. Other students may feel that how we behave as consumers and individuals plays a large, collective role in changing attitudes and behaviors necessary to create the political willpower for meaningful change.
      3. What can you do as a future physician or even today as a student?
         1. Incorporate environmental counseling into your patient care
            1. Acknowledging that the public health threat is real and well-evidenced is an important step. In 88,125 climate-related peer-reviewed publications since 2012, more than 99% found contemporary climate changes attributable to human activities.^10^
            2. Advise patients to minimize exposure to air pollution and excessive heat (be aware of Ozone Days, Heat warning days and the Air Quality Index)
            3. Consider patients’ social vulnerability as another non-medical driver of health in your holistic approach to their care
         2. Consider incorporating small conservation changes into your life
            1. Bike, walk and use public transportation whenever convenient
            2. Opt for plant-based dietary choices whenever possible to lessen the carbon effects of the meat/agriculture industry
            3. Consider electric vehicles, weatherize your home and opt for solar/wind-based energy plans
         3. Join advocacy efforts
            1. Enroll in or start environmental student electives. AMSA has a new national Environmental Health Action Committee to bring awareness to environmental justice and student advocacy.^[[12]](#endnote-12)^
            2. As with any healthcare issue, physicians can share their patients’ stories with elected officials to influence policymakers to better enforce of existing environmental regulations
         4. Exercise resource-mindfulness
            1. Subscribe to campaigns like Choosing Wisely that help minimize medical resource waste.^[[13]](#endnote-13)^ Participating organizations like the AAP, ACP, ACEP and ACS highlight unnecessary labs, imaging tests, and lengthy hospitalizations as ways to reduce costs and waste

*[10 minutes]* Take Home messages and **leave time for Post-Module survey**

1. **Take Home messages**
   1. Climate change is a well-evidenced threat to human health. There are impacts on nearly every organ system and implications for disease across many specialties. Educate yourself on which environmental determinants affect your patient population.
   2. As a community leader, please consider participating in conservation, advocacy and resource mindfulness.
2. **Optional Further Reading**
   1. [UN’s Intergovernmental Panel on Climate Change (IPPC): 6th Assessment Report](https://www.ipcc.ch/report/ar6/wg1/downloads/report/IPCC_AR6_WGI_SPM_final.pdf)^[[14]](#endnote-14)^ - This 2021 report summarizes the current state of the climate science, the definitive role of human influence, and policy recommendations to limit human-induced climate change.
   2. [CDC: Preparing for the Health Impacts of Climate Change in the Southern Great Plains](https://www.cdc.gov/climateandhealth/effects/SouthernGreatPlains.htm)^[[15]](#endnote-15)^ - This resource breaks down climate change’s effects (air, water, weather extremes, etc.) by region. Included is the Southern Great Plains region, but other regions have analogous sections.

1. How Climate Affects Community Health. Centers for Disease Control and Prevention. Updated Jul 9, 2019. Accessed July 6, 2022. <https://youtu.be/JywsWktvODc> [↑](#endnote-ref-1)
2. D'Amato G, Cecchi L, D'Amato M, Liccardi G. Urban air pollution and climate change as environmental risk factors of respiratory allergy: an update. *J Investig Allergol Clin Immunol*. 2010;20(2):95-102. [↑](#endnote-ref-2)
3. Salo PM, Arbes SJ Jr, Jaramillo R, et al. Prevalence of allergic sensitization in the United States: results from the National Health and Nutrition Examination Survey (NHANES) 2005-2006. *J Allergy Clin Immunol*. 2014;134(2):350-359. [↑](#endnote-ref-3)
4. Asthma Disparities in America: A Roadmap to Reducing Burden on Racial and Ethnic Minorities. Asthma and Allergy Foundation of America. Updated 2020. Accessed July 4, 2022. https://[www.aafa.org/asthmadisparities](http://www.aafa.org/asthmadisparities) [↑](#endnote-ref-4)
5. Today's Texas Air Quality Forecast. Texas Commission on Environmental Quality. Top of FormBottom of Form

   Updated July 7, 2022. Accessed July 7, 2022. <https://www.tceq.texas.gov/airquality/monops/forecast_today.html> [↑](#endnote-ref-5)
6. State of the Air: Health Impact of Air Pollution. American Lung Association. Updated 2022. Accessed July 7, 2022. <https://www.lung.org/research/sota/health-risks> [↑](#endnote-ref-6)
7. Lynas M, Houlton BZ, Perry S. Greater than 99% consensus on human caused climate change in the peer-reviewed scientific literature. *Environ Res Lett*. 2021;16:114005 [↑](#endnote-ref-7)
8. Climate change. World Health Organization. Updated 2022. Accessed on July 7, 2022. <https://www.who.int/health-topics/climate-change#tab=tab_1> [↑](#endnote-ref-8)
9. Texas Department of Health. https://www.dshs.texas.gov/chs/vstat/Hotcolddths/hotcolddths.shtm [↑](#endnote-ref-9)
10. Solar Industry Research Data. Solar Energy Industries Association. Accessed July 7, 2022. <https://www.seia.org/solar-industry-research-data> [↑](#endnote-ref-10)
11. Larsen K, Pitt H, Grant M, Houser T. China’s Greenhouse Gas Emissions Exceeded the Developed World for the First Time in 2019. Rhodium Group. Updated May 6, 2021. Accessed July 7, 2022. <https://rhg.com/research/chinas-emissions-surpass-developed-countries/> [↑](#endnote-ref-11)
12. Environmental Health Action Committee. American Medical Student Association. Updated 2022. Accessed July 7, 2022. <https://www.amsa.org/action-committee/environmental-health/> [↑](#endnote-ref-12)
13. Choosing Wisely. American Board of Internal Medicine Foundation. Accessed July 7, 2022. <https://abimfoundation.org/what-we-do/choosing-wisely> [↑](#endnote-ref-13)
14. Climate Change 2021: The Physical Science Basis. Intergovernmental Panel on Climate Change. Updated August 9, 2021. Accessed July 24, 2022. <https://www.ipcc.ch/report/ar6/wg1/downloads/report/IPCC_AR6_WGI_SPM_final.pdf> [↑](#endnote-ref-14)
15. Preparing for the Health Impacts of Climate Change in the Southern Great Plains. Centers for Disease Control and Prevention. Updated January 7, 2021. Accessed July 24, 2022. <https://www.cdc.gov/climateandhealth/effects/docs/Southern-Great-Plains_Regional-Climate-Fact-Sheet-P.pdf> [↑](#endnote-ref-15)
